# Fatal Human Co-infection with *Leptospira* spp. and Dengue Virus, Puerto Rico, 2010

**DOI:** 10.3201/eid1805.111555

**Published:** 2012-05

**Authors:** Tyler M. Sharp, Julio Bracero, Aidsa Rivera, Wun-Ju Shieh, Julu Bhatnagar, Irma Rivera-Diez, Elizabeth Hunsperger, Jorge Munoz-Jordan, Sherif R. Zaki, Kay M. Tomashek

**Affiliations:** Centers for Disease Control and Prevention, Atlanta, Georgia, USA (T.M. Sharp, W.-J. Shieh, J. Bhatnagar, S.R. Zaki);; Centers For Disease Control and Prevention, San Juan, Puerto Rico (T.M. Sharp, A. Rivera, E. Hunsperger, J. Munoz-Jordan, K.M. Tomashek);; Hospital Episcopal San Lucas, Ponce, Puerto Rico (J. Barcero);; Instituto de Ciencias Forenses Departamento de Patología, San Juan (I. Rivera-Diez)

**Keywords:** Leptospira spp., dengue virus, co-infection, dengue, dengue fever, dengue virus 1, DENV, leptospirosis, Puerto Rico, acute febrile illness, zoonoses, human, death, fatal, viruses, bacteria, dengue

**To the Editor:** Leptospirosis, caused by *Leptospira* spp. bacteria, and dengue, caused by dengue viruses (DENVs), are potentially fatal acute febrile illnesses (AFI) endemic to the tropics (*1**,**2*). Because their clinical manifestations are similar (*3*), leptospirosis may be misidentified as dengue (*4*). We report a fatal case of co-infection with *Leptospira* spp. and DENV-1 in a man in Puerto Rico.

On May 23, 2010, a 42-year-old unemployed male carpenter came to an outpatient clinic in Puerto Rico reporting a 4-day history of fever, headache, generalized myalgia, anorexia, nausea, and vomiting. He was being treated for chronic hypertension and had been released from jail 2 weeks before illness onset. On evaluation, he was febrile, hypertensive, and tachycardic; laboratory results showed thrombocytopenia and leukocytosis with a predominance of neutrophils. Viral syndrome was diagnosed, and the patient was given acetaminophen, solumedrol, and ketoprofen.

The patient returned to the clinic on May 25 with continued fever, myalgia, worsening headache, and bilateral calf pain; he was afebrile and tachycardic and appeared acutely ill. He had no rash, jaundice, icteric sclera, cardiac murmurs, or organomegaly, and his lungs were clear on auscultation. He was given intravenous (IV) saline, and results of laboratory tests performed afterward showed leukocytosis with a predominance of neutrophils, thrombocytopenia, increased blood urea nitrogen (BUN)–to-creatinine ratio, hyponatremia, hyperglycemia, and elevated aspartate aminotransferase. He was given IV ampicillin, meperidine, and promethazine and was transferred to a local hospital for admission, with a presumptive diagnosis of pre–renal azotemia and leptospirosis.

On arrival at the emergency department on the same day, the patient was febrile, tachycardic, and hypotensive, with cold, clammy skin. Results of an electrocardiogram showed sinus tachycardia; cardiac enzymes were not elevated. He was given repeat IV saline and piperacillin/tazobactam. New laboratory findings included anemia, prolonged prothrombin time, elevated creatinine kinase, hematuria, and a further increase in BUN-to-creatine ratio. Chest radiograph showed cardiomegaly with increased pulmonary vascularity and perihilar alveolar densities. Arterial blood gas (ABG) results showed compensated metabolic acidosis, with low oxygen partial pressure (pO_2_). He was given IV saline again, and vancomycin and ceftriaxone were added to his medication regimen.

On admission to the intensive care unit, the patient continued to be hypotensive and was again given IV saline. Although ABG results on the morning of May 26 were somewhat improved, the patient was started on respiratory treatments for new-onset cough and increasing respiratory rate. Laboratory test results showed a large drop in hematocrit, worsening thrombocytopenia and leukocytosis, hypocalcemia, and hypoalbuminemia; he was given an infusion of 25% albumin.

The patient’s condition continued to worsen, with ABG results showing further decline in pO_2_. Severe respiratory distress developed, and he was placed on mechanical ventilation and given IV saline. Repeat ABG results showed severe respiratory acidosis and metabolic acidosis. Soon after, generalized edema developed, and the patient became cyanotic, with no measurable pulse; despite aggressive resuscitation efforts, he died on March 26.

All results of bacterial cultures were negative, as was detection of anti-*Leptospira* IgM. Postmortem examination showed rash and pleural effusion, and blood and tissue specimens were taken for diagnostic testing. Liver sections showed bile stasis, dilated sinusoidal space, and pericentral hepatocellular necrosis (Figure, panel A); lung sections showed intraalveolar hemorrhage, edema, and focal inflammatory infiltrates (Figure, panel B). Heart sections showed perivascular edema, and kidney sections showed evidence of interstitial inflammatory infiltrates and acute tubular necrosis (Figure, panel C). Immunohistochemical analysis of kidney (Figure, panel D), liver, lung, and heart sections showed *Leptospira* antigen. Dengue virus nonstructural (NS) protein 1 was detected in whole blood, and flavivirus NS5 gene was amplified from RNA extracted from the liver; sequencing showed 98% homology with DENV-1.

**Figure:**
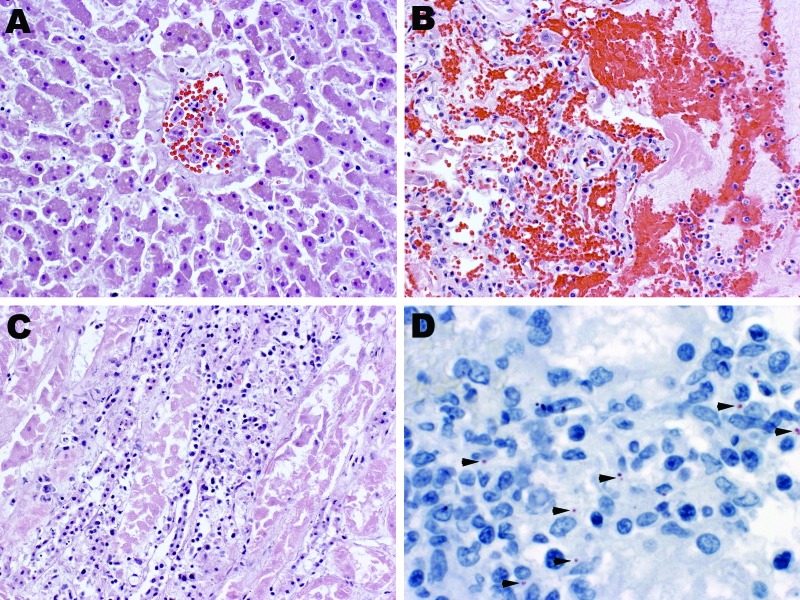
Histopathologic evaluation of tissue samples collected postmortem from a person co-infected with *Leptospira* spp. and dengue virus 1. Tissue specimens were taken from the liver (A), lung (B) and kidney (C and D) and stained with hemotoxylin-eosin (A, B, C; original magnification ×20) or probed with poly clonal anti-*Leptospira* antibody for immunohistochemical detection of *Leptospira* antigen (D; arrowheads indicate antigen; original magnification ×63).

This case report demonstrates the need for antigen-based rapid diagnostic tests (RDT) for AFI patients. All available leptospirosis RDTs detect anti-*Leptospira* IgM (*5*), which was not detectable in this patient’s blood on the seventh day of illness, although *Leptospira* antigen was detected in postmortem analysis. Therefore, it is unlikely that any available leptospirosis RDT would have been clinically useful when leptospirosis signs first were recorded on the fourth day of illness. Because the incidence of both dengue and leptospirosis is increasing worldwide (*6**,**7*), physicians should have access to antigen-based RDT to make timely and thorough diagnoses.

Nonetheless, even if leptospirosis had been diagnosed in this patient, dengue virus infection would likely still have been overlooked. Therefore, clinicians in areas where both *Leptospira* spp. and DENVs are endemic should include both pathogens in the differential diagnosis when evaluating AFI patients and should consider the possibility of co-infection. Early administration of doxycycline and penicillin G to treat mild and severe leptospirosis, respectively, may reduce the duration and severity of illness (*8*). For cases of severe dengue, packed red blood cells should be given in response to severe anemia. For patients with either dengue or leptospirosis, intravenous fluid administration should be closely monitored to prevent fluid overload.
